# Impact of High Intensity Contact Physical Activity During a Match on Echocardiographic Parameters and High-Sensitivity Troponin I in Competitive Rugby Players

**DOI:** 10.3390/jcm14072226

**Published:** 2025-03-25

**Authors:** Petra Radic, Nikola Bulj, Sinisa Car, Martina Cancarevic, Aljosa Sikic, Diana Delic-Brkljacic, Marin Pavlov, Zdravko Babic

**Affiliations:** 1 Department of Cardiology, Sestre Milosrdnice University Hospital Centre, 10000 Zagreb, Croatia; 2 School of Medicine, University of Zagreb, 10000 Zagreb, Croatia; 3 Department of Emergency Medicine, Sestre Milosrdnice University Hospital Centre, 10000 Zagreb, Croatia; aljosa.sikic@kbcsm.hr; 4 Department of Cardiology, University Hospital Dubrava, 10000 Zagreb, Croatia

**Keywords:** rugby, physical activity exposure, troponin, echocardiography

## Abstract

**Background/Objectives**: High-intensity physical activity, especially in contact sports, may have harmful effect on athletes’ health. The aim of the study is to determine acute changes in the function of the left ventricle in rugby players after a competitive match. **Methods**: A prospective, clinical, observational case–control study was conducted. All cases were European Caucasian male athletes, older than 18 years, who had played for at least 60 min in the investigated match. A transthoracic echocardiography and blood tests were performed in all the participants two days before the match and within one hour after the match. **Results**: The total number of examinees was 34. Out of the 31 measured echocardiographic parameters, 22 showed a statistically significant change before and after the match. We also observed changes in echocardiographic parameters in relation to the increase in high-sensitivity troponin I. Two-dimensional left ventricle (LV) end-systolic (75 ± 10.5 vs. 67.1 ± 10 mL, *p* = 0.032) and LV end-diastolic (149.7 ± 24.6 vs. 133.8 ± 13.3 mL, *p* = 0.020) volumes, 3D LV end-systolic volume (75.8 ± 9.2 vs. 67.4 ± 9.5 mL, *p* = 0.014), indexed 2D LV end-diastolic volume (67.6 ± 9.3 vs. 61.4 ± 8 mL, *p* = 0.042), and indexed 3D LV end-systolic (34.3 ± 3.8 vs. 31 ± 4.8 mL, *p* = 0.033) volume after the match were significantly higher in players with troponin increase. **Conclusions**: High-intensity contact activity, such as rugby, leads to acute changes in echocardiographic parameters, especially in athlete’s who experience elevation in troponin.

## 1. Introduction

Daily physical activity has been proven to be a beneficial factor in the prevention of numerous diseases, particularly cardiovascular diseases, and has been associated with a reduction in mortality [1,2].

Evidence suggests that 150 min of moderate-intensity or 75 min of vigorous-intensity aerobic exercise per week of leisure-time physical activity (LTPA) can have significant health benefits [3].

However, there is a threshold beyond which excessive physical activity can become harmful. One of the important goals of sports medicine is to determine the dose–response curve between exercise and health risk at prominent levels of intensity and duration. Several studies have highlighted biochemical, echocardiographic, and clinical evidence of cardiac dysfunction and injury, as well as cardiac disorders in active and recreational athletes [4,5].

Rugby is a high-intensity sport, with moderate static (10–20%) and moderate dynamic (50–75%) components [6]. The combative nature of the sport combined with the intermittent high-intensity physical activity may cause repetitive blunt-force injuries, especially to the chest.

Cardiac troponin is a biomarker of heart muscle injury used to diagnose acute coronary syndrome [7]. An increase in troponin levels in healthy individuals after physical activity has been previously described [8]. It is considered a consequence of physiological, not pathological, processes, although the mechanism behind this phenomenon has not yet been fully clarified [9]. It is speculated that an increase in the permeability of the myocyte membrane induced by mechanical stress, the production of oxidative radicals, and preload-induced increase in stretch-responsive integrins may be the basis for exercise-induced troponin increase [9].

The clinical significance of intensive physical activity and frequent thoracic injuries in contact sports, such as rugby, on myocardial function has not been fully clarified. To the best of our knowledge, echocardiographic parameters and high-sensitivity troponin I (hs-TnI) levels in rugby players before and after a match have not yet been investigated. The aim of this study was to describe the changes in echocardiographic and hs-TnI in a cohort of high-level rugby players before and after a competitive rugby match.

## 2. Materials and Methods

### 2.1. Participants Population

A prospective, clinical, observational case–control study was conducted. Only players engaged in the First Croatian Rugby League were included. All participants were European Caucasian athletes. Inclusion criteria were as follows: players older than 18 years, who played for at least 60 min in the investigated match, capable of understanding and signing an informed consent.

The research was carried out as follows: at least two days before scheduled competitive match all participants underwent vital signs measurement, ECG recording, transthoracic echocardiography, and blood tests in the Sestre Milosrdnice University Hospital Centre. The same procedure was repeated within one hour after the end of the match in the Sestre Milosrdnice University Hospital Centre. All exams were performed between April and May 2023.

### 2.2. Data Collection

We conducted history-taking with each participant. We asked about demographic information (age, level of education, and employment status), information related to rugby (years of training and position in the game), relevant medical histories (personal and familial), smoking habits, and alcohol consumption. We measured their height and weight, chest circumference, both hands grips, and vital signs (blood pressure, pulse, respiratory rate, oxygenation, and body temperature). We calculated their body mass index and body surface area. Transthoracic echocardiography data included standardized measurements recommended by both the European Association of Echocardiography and the American Society of Echocardiography [10,11]. We also recorded an electrocardiogram of each participant before and after the game and analyzed it. The laboratory marker that was used was high-sensitivity troponin I.

### 2.3. Blood Tests

Venous blood samples from the antecubital vein were collected in test tubes (13 mL in total) on the day of the first exam and within one hour after the end of the match. Complete blood count, biochemical profile including hs-TnI, as well as coagulation tests were measured.

The blood for hs-TnI measurement was allowed to clot at room temperature and was then centrifuged at 3500× *g* for 15 min. The method for determining hs-TnI concentration in a serum sample consisted of two steps using chemiluminescence microparticle immunoassay (CMIA) technology.

In the first step, the sample was mixed with diluent and paramagnetic microparticles coated with anti-troponin I antibodies. Troponin I present in the sample bounds to the microparticles. After incubation and washing, acridinium-labeled anti-troponin I conjugate was added. After incubation and washing, trigger and pre-trigger solutions were added to the reaction mixture, after which chemiluminescence was measured. The resulting chemiluminescence was measured in relative light units. The concentration of hs-TnI was then determined from the curve obtained using a calibrator with known concentrations of hs-TnI. Values considered to be elevated were >16 ng/L.

### 2.4. Echocardiography

All echocardiographic examinations were performed using the same ultrasound machines (GE Vivid E95, General Electric, Milwaukee, WI, USA). Standard views of the heart were obtained with a 2.8 MHz frequency phased-array transducer. All reviews were stored in digital archive form for subsequent analysis (off-line postprocessing analysis). Each analysis was performed by a certified and experienced operator using European Association of Echocardiography recommendations for standardization of performance, digital storage, and reporting of echocardiographic studies [10]. Postprocess analysis was performed using the GE EchoPAC v206 software, within which automated functions for measuring target echocardiographic values were used. All absolute values were indexed to body surface area (BSA). All examinations were performed at tentatively the same time of day to avoid possible circadian variations in echocardiographic parameters.

All procedures were performed in accordance with ethical standards of the Helsinki Declaration and were approved by the Ethics Committee of Sestre Milosrdnice University Hospital Centre. All participants signed an informed consent to the investigators before participating.

All matches were recorded with a video camera, and the number of tackles and blows to the chest of an individual player was counted using the official video of the observed match.

### 2.5. Study Endpoint

The primary aim of the endpoint was to determine whether high-intensity exercise, such as participation in a competitive rugby match, has an acute effect on the echocardiographic parameters of rugby players and whether there is a change in levels of hs-TnI. A secondary aim was to determine whether there are correlations between the two.

### 2.6. Statistical Analysis

The normality of distribution was tested using a Kolmogorov–Smirnov test. Descriptive variables are presented as frequencies (percentages), means (standard deviations) or medians, and interquartile ranges (IQRs). A paired-samples T-test or Wilcoxon signed-rank test were used to compare variables determined before and after the match. Pearson’s or Spearman’s correlation coefficients were used to test the strength of inter-variable correlations. Binary logistic regression was used to determine variables independently associated with the increase in hs-TnI after the match (study population divided into players with and without an increase in troponin after the match). A receiver operating characteristic (ROC) curve analysis was used to determine the predictive power of echocardiographic variables on troponin increase after the match. The significance tests were two-tailed, and *p* < 0.05 was considered significant. Statistical analyses were performed with SPSS for Windows, version 25 (IBM, Armonk, NY, USA).

## 3. Results

A total of 37 professional rugby players were screened. After an initial transthoracic echocardiography, three players were excluded from the study. In one case, a hypertrophic myocardium with significant thickening of the septum was verified, and further work-up and treatment was recommended. In the remaining two players, a diagnosis of bicuspid aortic valve was established. One was classified as having severe aortic regurgitation, requiring surgical treatment. In the second player, the bicuspid valve function was normal. Further clinical monitoring was recommended. Thus, a total of 34 professional rugby players were enrolled in the study. The median age was 26 (22–30) years. All study subjects were male. General characteristics, vital signs, and electrocardiographic parameters are presented in Table 1.

The analysis of echocardiographic parameters is presented in Table 2. Median ejection fraction before the match calculated using two-dimensional (2D) and three-dimensional (3D) methods were similar 54.5% (50–66%) and 54% (45–67%) respectfully. Out of 31 echocardiographic variables, 22 were significantly different when compared before and after the match (Table 2).

**Figure 1:**
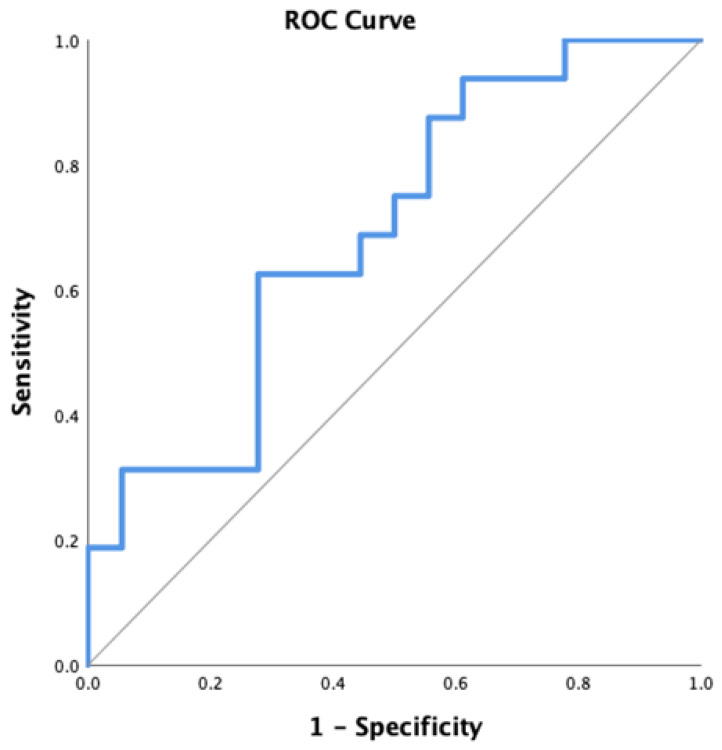
ROC analysis of the predictive power of end-diastolic 2D volume after the match on hsTnI increase.

Initial hs-TnI was under the detection limit in all players. After the match, hs-TnI increased above the level of detection in 16 (47.1%) players. The average level of hs-TnI in players with hs-TnI elevation was 21 (14–45) ng/L. The study population was then divided into two groups according to the increase in the troponin level (not present or present), and all analyzed echocardiographic variables were compared between the two groups. Two-dimensional left ventricle (LV) end-systolic (75 ± 10.5 vs. 67.1 ± 10 mL, *p* = 0.032) and LV end-diastolic (149.7 ± 24.6 vs. 133.8 ± 13.3 mL, *p* = 0.020) volumes, 3D LV end-systolic volume (75.8 ± 9.2 vs. 67.4 ± 9.5 mL, *p* = 0.014), indexed 2D LV end-diastolic volume (67.6 ± 9.3 vs. 61.4 ± 8 mL, *p* = 0.042), and indexed 3D LV end-systolic (34.3 ± 3.8 vs. 31 ± 4.8 mL, *p* = 0.033) volume after the match were significantly higher in players showing a troponin increase. To analyze the independent association with hs-TnI increase following the match, a binary logistic regression with all five previously mentioned echocardiographic variables was performed (backward approach). End-diastolic 2D volume after the match was a single variable significantly associated with hs-TnI increase (exp(b) = 1057, 95% confidence interval 1.002–1.114, *p* = 0.041; the variable explains 22.7% of the model variability). ROC analysis was performed to further detect the predictive power of end-diastolic 2D volume after the match on troponin increase. The acquired area under the curve was 0.691 ±0.091 (95% CI 0.513–0.869), *p* = 0.058 (Figure 1).

**Figure 2:**
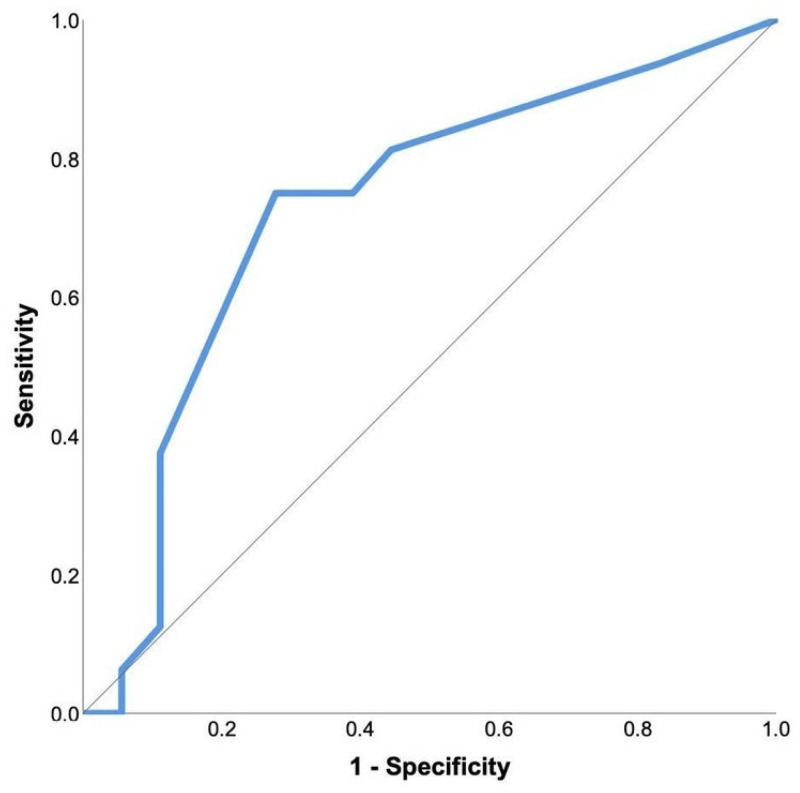
ROC analysis of the predictive power of the number of blows to the chest during the match on hs-TnI increase.

Echocardiographic variables were analyzed in regard to the parameters of physical contact (number of tackles, blows to the chest). Number of blows to the chest and tackles did not significantly correlate with any echocardiographic parameter changes. In a ROC curve analysis of the number of chest hits in relation to troponin elevation, a significant predictive value was detected (AUC 0.727, 95% CI 0.549–0.906; *p* = 0.024) (Figure 2). By using the Youden index, 4.5 chest hits were determined as the cut-off value to predict the increase in troponin levels, with a sensitivity of 75% and specificity of 72%.

## 4. Discussion

In this study, high-intensity contact physical activity, such as active participation in a competitive rugby match, was associated with significant changes in echocardiographic parameters, especially in athletes who experienced an elevation in hs-TnI levels.

Chronic endurance training may lead to a series of morphological and physiological adaptations of the myocardium, referred broadly to as athlete’s heart [12]. Although the benefits of long-term physical activity are often emphasized, the acute effects of vigorous training on myocardial function have not been fully elucidated. Studies are recently focusing on the impact of prolonged high cardiac workload on transient dysregulation of cardiac function. The phenomenon has been referred to as “exercise-induced cardiac fatigue” (EICF) [13].

EICF is defined as an acute reduction in the systolic and diastolic function of the left ventricle after exercise as a consequence of the decrease in the contractile and filling properties of the left ventricle [13]. EICF has not been consistently detected in all studies focusing on myocardial function in athletes, which may be due to a great diversity in the dynamic and static components of a particular sport, as well as variability in the duration of physical activity.

Middleton et al. performed a meta-analysis on 34 studies focusing on left ventricular function after prolonged exercise [14]. The results demonstrate a significant overall decrease in left ventricle ejection, suggesting a potentially transient decrease in systolic function, a fact consistent with our study. The downregulation of beta-adrenergic receptors following prolonged exposure to increased levels of circulating catecholamines may be the culprit behind the reduction in LV contractility [15].

Similarly, a reduction in the E/A value after physical exertion was detected, both in previous studies and in ours. Study participants, with initially normal transmitral diastolic filling at rest, developed an increased share of atrial contribution to LV filling volume post-exercise associated with a shortening in isovolumic relaxation. This finding suggests that atrial systole is an important contributor to cardiac reserve during exercise [16].

The data on dynamics of the E/E′ ratio in athletes after exposure to physical exertion are scarce. The value of E/E′ is considered a useful parameter for the non-invasive assessment of left ventricular filling pressure and left ventricular diastolic function. In a meta-analysis, E/E′ ratio was found to not be suitable for estimating diastolic function in response to exercise. However, aside from the fact that the majority of the studies were performed in a single center with a low sample size and heterogeneity of the methodology, hindering the data usability [17]. In our research, an elevation in the E/E′ value was recorded, but it was not statistically significant.

Our data suggest that, in addition to the decrease in left ventricle ejection fraction, there is also a decrease in the LV GLS. As physical activity has a positive effect on cardiovascular health, it may be assumed that LV GLS increases as a result of training. Murray et al. performed a meta-analysis with the aim of verifying the effects of exercise on LV GLS in healthy individuals, as well as those with cardiovascular diseases [18]. Interestingly, the authors found that, in healthy individuals, exercise had a negative or no effect on LV GLS values compared to the control group. This is consistent with the findings in our study. The mechanism of the effect of physical activity on LV GLS values is still unclear, as well as the required duration of activity, leading to significant changes. The question of the longevity of the mentioned GLS changes persists, as well as the time required for the values to return to the initial levels. By reviewing the literature, we found that this is the only study that observed the effect of high-intensity contact physical exercise on a variety of echocardiographic parameters, among which was LV GLS.

When discussing the possible causes of changes in echocardiographic parameters and increased hsTn-I values observed in our study, we should also consider factors such as oxidative stress, inflammation, and mechanical stress on the myocardium, which have been shown to have an impact on myocardial function.

The oxidative stress state is characterized by an increase in the amount of reactive oxygen and nitrogen species that overwhelms the antioxidant defense systems of the body. Physical activity leads to an increase in the oxygen demand of the body and, consequently, to the development of oxidative stress. Evidence from the literature supports the involvement of oxidative stress in the pathophysiology of numerous cardiovascular diseases such as congestive heart failure, cardiac hypertrophy, and myocardial post-infarction remodeling.

Arterial hypertension is an important risk factor for the development of cardiovascular diseases [19], and oxidative stress is central to its development. Rodrigo et al. observed, in their study, an increase in the production of hydrogen peroxide and superoxide and the reduction in the synthesis of nitric oxide, which leads to arterial hypertension [20]. Moreno Ruiz et al. demonstrated a positive correlation between oxidized proteins and GLS. In patients with arterial hypertension who had elevated levels of oxidized proteins, lower values of longitudinal myocardial deformation were measured, as well as the lower systolic ejection function of the left ventricle [21]. Accordingly, we could use the same analogy to explain the decrease in LVEF and LV GLS observed in our study after exposure to high-intensity physical activity.

It has been shown that moderate levels of exercise-induced oxidative stress favors the adaptation of skeletal muscle. However, high levels of radical oxygen species (ROS) production results in damage to the muscle and a decline in the physiological benefits associated with low to moderate levels of ROS production [22]. The above refers to the previously mentioned theory that there may be an upper limit to physical activity that is beneficial to the human body. However, all prior research related to physical activity and oxidative stress focused on skeletal muscles, not the myocardium. Oxidative stress has a proven role in cardiac remodeling in patients with cardiovascular diseases, but additional research is needed to determine whether a certain form or level of physical activity can be a precursor for the development of certain cardiac conditions.

Inflammation is closely related to oxidative stress. An excess of reactive oxygen species due to oxidative stress triggers signaling cascades that can lead to the onset and progression of inflammation. Several conditions, pathological and physiological, responsible for damage or death of myocardial cells, among which is inflammation, cause an increase in troponin levels in the absence of myocardial necrosis [23]. At the same time, chronic inflammation is also a proven risk factor for the development of heart failure through mechanisms such as microvascular dysfunction and endothelial function dysregulation, leading to fibrosis and diastolic dysfunction [24].

As the sensitivity of troponin assays has increased, specificity has decreased, leading to more frequent cases of patients with elevated troponin levels in the absence of cardiovascular disease [25]. The exact mechanisms of troponin release from cardiomyocytes into the bloodstream after physical activity remain unclear [26]. According to recent findings, there are four possible mechanisms of troponin release after acute physical activity [27]. First, physical activity increases the cardiomyocyte cell membrane permeability [28], and it is assumed that small unbound cytosolic troponin fragments can subsequently leak into the circulation via cell wounds, cytoplasmic bleb formation, or exocytosis. Second, research on mice showed that eight weeks of monitored wheel running could increase the rate of cardiomyocyte turnover [29], and an acute bout of vigorous exercise might accelerate troponin release from replaced cardiomyocytes into circulation. Third, increases in preload [30] and brief ischemia [31], both present in high-intensity physical activities, can increase the rate of apoptosis. Fourth, ischemia is present in vigorous physical activity, and it may induce necrosis. Despite the above, myocardial edema or late gadolinium enhancement were not present in marathon runners despite significantly elevated post-exercise troponin concentrations [32].

The significance of elevated cardiac biomarkers after endurance exercise remains unclear. In some studies, an increase in serum troponin values after exposure to physical effort occurs in 23–47% of subjects [33], which correlates with the results of our study.

Several researchers have attempted to identify predictors of exercise-induced troponin release to explain the mechanisms of troponin release in athletes. In their study, Aengevaeren et al. ranked age, sex, body composition, exercise intensity and duration, training experience, hydration status, environmental factors, cardiovascular health status, and blood pressure among the predictors of troponin release [27]. Factors that affect the amount of cardiac workload, such as exercise intensity and duration, appear to have the most significant effect [34]. However, the predictive value of all these factors on post-exercise troponin concentrations remains low (r2 < 35%) [35]. Consequently, it is presumable that there are internal and external factors that affect the magnitude of exercise-induced troponin elevations. A meta-analyses compared troponin release following different exercise modes [33]. Nevertheless, it is still largely unknown how the type of exercise affects the concentrations of troponin because there have been no controlled studies that would contribute to the clarification of this question. Still, in marathon runners [36], larger exercise-induced troponin elevations were recorded compared with long-distance walkers [37] covering a comparable distance [38], indicating that exercise mode does impact troponin release to some extent.

Recent evidence also suggests that skeletal muscle injury might also cause an increase in troponin concentration [39]. It should also be noted that troponin elevations may occur in up to 30% of cases during rhabdomyolysis, a frequent consequence of vigorous sports, without any signs of cardiac injury [40]. Lippi et al. conducted research based on a model of eccentric exercise, a type of muscular activity sufficient to evoke skeletal but not myocardial muscle injury, skeletal muscular injury led to an increase in concentration of troponin T, up to 13% [41]. The contribution and interference of skeletal muscle damage should not be overlooked when commenting on changes in troponin level concentrations.

Our data suggest that athletes with an increase in hs-TnI have more significant changes in certain echocardiographic parameters. More significant trauma of the myocardium may be behind this phenomenon, considering that, in a rugby match, athletes receive a number of blows to the chest. Multiple bouts of mechanical stress forces, such as in a rugby match, may elicit stretching of the myocytes, underpinning an inflammatory reaction [42]. Repetitive episodes of mechanical stress combined with elevations in troponin, followed by immediate restoration of normality, may represent a physiological repairing or even remodeling process. During mechanical stress on muscles, including the myocardium, a process called mechanotransduction occurs, whereby cells transduce mechanical forces to chemical and electrical responses. Mechanotransduction is required for balancing cell and tissue structure and function. In vitro studies have been widely used to study how mechanical forces impact myocyte structure and function, but it has not yet been clarified how these processes function in in vivo models. Mechanotransduction is especially complex in the myocardium because of the numerous types of dynamic mechanical loads applied to myocytes. Additional research is needed to determine the possible long-term effects of mechanical stress accumulated during high-intensity physical activity on athletes’ cardiovascular health.

Rugby players are exposed to frequent injuries, including blunt chest injuries. The minimal amount of protective equipment on the chest also contributes to frequent injuries [43]. Research regarding injury surveillance during International Rugby Board Rugby World Cup (IRBRWC) 2007 established that 8.3/1000 player-hours were lost from match injuries involving the upper back/sternum/ribs. A study revealed that tackling and being tackled posed the greatest injury burden during rugby matches [44]. Our results suggest that tackles do not significantly correlate with any echocardiographic parameter or elevation of hs-TnI, but, on the other hand, our data show that we can expect hs-TnI elevation in players who receives five or more blunt blows to the chest.

This observation could be explained with the definition and performance of tackles. The tackle is made below the sternum so that the tackler uses arms to grab and hold the ball carrier. If the ball carrier is lifted off the ground, they are brought back to the ground safely and the tackler then releases the tackled player. On the other hand, during the game, if a blunt chest trauma occurs, most often we witness direct blows to the chest area, where rugby players have no protective equipment. Several case reports have been published in the literature about rugby players who developed acute myocardial infarction due to coronary artery dissection [45] or aortic rupture [46] after blunt chest trauma. Research using sensors designed to detect and measure force, acceleration, and impact experienced during athletic activities could help shed light on just how dangerous impacts are during rugby matches.

Our study adds multiple insights into the potential cardiovascular side-effects of high-intensity contact physical activity, such as rugby. Studying cardiac parameters before and after a rugby match provides information on cardiac response to high-intensity intermittent exercise and blunt chest trauma. Future studies may detect the potential risk factors associated with cardiac stress during rugby, such as the frequency and intensity of high-intensity bursts, impact from tackles and chest blows, and duration of the game.

We would also like to emphasize the need for adequate sports examinations, especially for athletes who participate in high-intensity sports, such as rugby. In our study with a relatively small number of subjects, we found that as many as three pathological transthoracic echocardiography findings in athletes who regularly received permits for professional sports participation twice a year. Our cohort confirms the need for the rigorous screening of athletes for heart murmurs and ECG abnormalities and subsequent transthoracic echocardiography.

Information from studies such as ours may be crucial for player safety and guide recommendations for training and competition. These findings may contribute to the development of more informed training and recovery protocols for rugby players. This could include recommendations for cardiovascular conditioning, proper warm-up routines, and strategies to minimize the risk of cardiac events during and after a game. This study could contribute to the establishment of guidelines for player safety, including recommendations for pre-participation screening such as an echocardiographic exam, monitoring, and medical support during games. Another important issue to discuss in the future is the need for development of protective equipment to reduce chest trauma and thus long-term consequences on the athlete’s cardiovascular system. Finally, our study contributes to the broader field of sports science, enhancing our understanding of the cardiovascular adaptations to a specific sports. This knowledge could have implications beyond rugby, potentially influencing training strategies in other sports with similar demands.

### Strengths and Limitations of the Study

To date, this is the only study that has looked at echocardiographic parameters and hs-TnI in rugby players before and after a competitive match.

There are also several limitations to the present study. The study population was relatively small. Ethnicity and sex may be an important factor in such a study. Only male European Athletes were included. Absence of long-term follow-up prevents the analysis of the long-lasting effects of high-intensity contact physical activity, such as rugby, on myocardial function.

## 5. Conclusions

In light of the already published data, our study provides further evidence that high-intensity exercise, in this case rugby, has acute effects on echocardiographic parameters, especially on LV ejection function and GLS. hs-TnI proved to be a strong predictor of more significant changes in echocardiographic parameters and therefore might improve the stratification of players who are at a higher risk of myocardial injury. Nevertheless, additional research is needed to investigate the long-term effects of highintensity contact physical activity on the myocardium of athletes.

## Figures and Tables

**Table 1:** General characteristics, vital signs, and electrocardiographic parameters of investigated rugby players.

	Percentage (%), Mean (Standard Deviation) or Median (Interquartile Range)	
Age	26 (22–30)	
Smokers	23.52%	
Duration of smoking (years)	9 (6–13)	
Number of cigarettes per day	13 (10–18)	
Alcohol consumption	94.11%	
Level of education (higher education/secondary education)	67.64%/32.35%	
Employment status (unemployed/student/employed)	11.76%/32.35%/55.88%	
Chronic therapy †	5.88%	
Duration of rugby training (years)	13 (9–15)	
Forward pack/back line	47.05%/52.94%	
Height (cm)	182 (175–186)	
Mass (kg)	96.2 (85.4–110.9)	
BMI (kg/m^2^)	28.9 (26.3–33.1)	
BSA (m^2^)	2.2 (0.2)	
Chest circumference (cm)	107 (10)	
Right hand grip (kg)	40.6 (9)	
Left hand grip (kg)	36.8 (10.5)	
Systolic pressure before the match	Before the match (mmHg)	134 (16)
	After the match (mmHg)	132 (12)
Diastolic pressure	Before the match (mmHg)	76 (12)
	After the match (mmHg)	75 (11)
Pulse	Before the match (/min)	72 (10)
	After the match(/min)	92 (11)
Respiratory rate	Before the match (/min)	16(14–18)
	After the match (/min)	22 (18–24)
Oxygen saturation	Before the match (%)	98 (97–98)
	After the match (%)	98 (97–98)
Body temperature	Before the match (°C)	36.4 (36.2–36.5)
	After the match (°C)	36.4 (36.3–36.6)
PR interval	Before the match (ms)	156 (25)
	After the match (ms)	155 (19)
QRS duration	Before the match (ms)	97 (9)
	After the match (ms)	96 (8)
QT duration	Before the match (ms)	398 (25)
	After the match(ms)	360 (22)
QTc	Before the match (ms)	397 (390–409)
	After the match (ms)	402.9 (12.4)

† Levothyroxine. Legend: BMI—body mass index; BSA—body surface area. QTc—corrected QT interval.

**Table 2:** Descriptive values of echocardiographic parameters before and after the match.

	Mean (Standard Deviation) or Median (Interquartile Range)		
	Before the Match	After the Match	*p*
LVEF 2D (%)	55 (51–57)	50 (47–52)	<0.001
LVEF 3D (%)	55 (5)	50 (4)	<0.001
LV diameter (mm)	51 (4)	53 (4)	<0.001
Indexed (BSA) LV diameter (mm/m^2^)	23.2 (2.1)	24 (2)	<0.001
E (cm/s)	0.77 (0.11)	0.66 (0.11)	<0.001
A (cm/s)	0.5 (0.09)	0.52 (0.1)	0.269
E/A	1.48 (1.36–1.82)	1.26(1.18–1.41)	<0.001
E′ septal (m/s)	0.12 (0.02)	0.1 (0.02)	<0.001
E′ lateral (m/s)	0.18 (0.03)	0.15 (0.03)	<0.001
Mean E′ (m/s)	0.15 (0.02)	0.13 (0.02)	<0.001
E/E′ mean	5.3 (0.84)	5.37 (1.12)	<0.001
2D LV volume (end-systolic) (mL)	64 (8)	71 (11)	0.004
2D LV volume (end-diastolic) (mL)	140 (131–157)	138 (129–149)	0.487
3D LV volume (end-systolic) (mL)	64 (9)	71 (10)	<0.001
3D LV volume (end-diastolic) (mL)	142 (20)	145 (20)	0.126
Indexed 2D LV volume (end-systolic) (mL/m^2^)	29.4 (3.6)	32.3 (4.9)	0.005
Indexed 2D LV volume (end-diastolic) (mL/m^2^)	65.3 (8.1)	64.3 (9.1)	0.479
Indexed 3D LV volume (end-systolic) (mL/m^2^)	29 (4.5)	32.5 (4.6)	<0.001
Indexed 3D LV volume (end-diastolic) (mL/m^2^)	64.7 (8.5)	66.22 (8.2)	0.174
LV GLS (%)	−17.9 (1.6)	−16 (1.7)	<0.001
2D stroke volume (mL)	78 (70–89)	70 (62–75)	<0.001
3D stroke volume (mL)	77 (67–85)	73 (65–82)	0.001
Indexed 2D stroke volume (mL/m^2^)	36.1 (33.2–40.2)	32 (29.1–35.3)	<0.001
Indexed 3D stroke volume (mL/m^2^)	36 (6.8)	33.29 (5.5)	0.001
Basal septum TVI (%)	−20.1 (6.1)	−13.9 (6.2)	<0.001
Mid-septum TVI (%)	−14.5 (6)	−10.3 (−13.6–−7.9)	0.006
Apical septum TVI (%)	−20.1 (5.4)	−16.4 (5.6)	0.003
Basal lateral TVI (%)	−13.4 (6.9)	−8.8 (4.7)	<0.001
Mid-lateral TVI (%)	−12.4 (6.2)	−12 (6.5)	0.782
Apical lateral TVI (%)	−12.5 (7.7)	−12 (7.5)	0.601
Mean TVI (%)	−15.5 (2.4)	−12.3 (2.7)	<0.001

Legend: BSA—body surface area; LV—left ventricle; LV GLS—left ventricle global longitudinal strain; LVEF—left ventricle ejection fraction; TVI—tissue velocity imaging; 2D—two-dimensional; 3D—three-dimensional.

## Data Availability

The datasets used and/or analyzed during current study are available from the corresponding author on reasonable request.

## Abbreviations

The following abbreviations are used in this manuscript:

hs-TnI	high-sensitivity troponin I
CMIA	chemiluminescence microparticle immunoassay
ECG	electrocardiogram
ROC	receiver operating characteristic
ROS	radical oxygen species
2D	two-dimensional
3D	three-dimensional
LV	left ventricle
GLS	global longitudinal strain
